# Assessment of Cognitive and Motor Skills in Parkinson's Disease by a Robotic Object Hitting Game

**DOI:** 10.3389/fneur.2019.00019

**Published:** 2019-01-28

**Authors:** Winnugroho Wiratman, Shunsuke Kobayashi, Fang-Yu Chang, Kohei Asano, Yoshikazu Ugawa

**Affiliations:** ^1^Department of Neurology, School of Medicine, Fukushima Medical University, Fukushima, Japan; ^2^Department of Neurology, Faculty of Medicine, Universitas Indonesia, Jakarta, Indonesia; ^3^Kokoro Research Center, Kyoto University, Kyoto, Japan; ^4^Department of Neuro-Regeneration, Fukushima Medical University, Fukushima, Japan; ^5^Department of Neurology, Aizu Chuo Hospital, Fukushima, Japan

**Keywords:** Parkinson's disease, virtual reality, visual discrimination, executive abilities/function, kinematics, cognitive performance, levodopa, decision-making

## Abstract

Parkinson's disease (PD) patients experience various symptoms including extrapyramidal motor disturbances and cognitive impairments, which cause difficulties in daily life. However, PD patients have rarely been studied under realistic task situations that require high-level interaction of cognitive and motor skills. The aim of this study was to investigate the contribution of cognitive and motor factors to the performance of PD patients under high cognitive and kinematic loads. Twenty-six PD patients and 14 control subjects participated in the study. The PD patients performed a task involving hitting targets and avoiding distractors in levodopa On and Off states. A robotic manipulandum device recorded the numbers of target and distractor hits and hand kinematics, including movement area and speed. Performance on standard cognitive batteries and the Movement Disorder Society – Unified Parkinson's Disease Rating Scale motor scores were examined. The results indicated that the PD patients hit significantly fewer targets and more distractors than did the controls (*p* < 0.05). In PD patients, the average hand speed was slower and the area of hand movement was smaller than those of the control subjects (*p* < 0.001). Levodopa significantly increased the average hand speed and movement area (*p* < 0.01), but levodopa had an insignificant effect on the number of correct targets hit and erroneous distractor hits. The scores of cognitive batteries predicted the performance with regard to both targets hit and distractor avoidance. Our results were indicative of a dynamic interaction between cognitive and kinematic skills while the PD patients performed a virtual reality game. Single-dose levodopa enhanced kinematic capacity, and the global intelligence level predicted game performance.

## Introduction

Parkinson's disease (PD) is a neurodegenerative disease that manifests cardinal motor symptoms of bradykinesia, rigidity, resting tremor, and postural instability. Recently, clinicians have become increasingly aware of non-motor symptoms of PD. Muslimovic et al. (1) reported that approximately 25% of *de novo* PD patients present with cognitive deficits. The most frequently impaired cognitive domains are attention, visuospatial, and executive functions (1–3). Varalta et al. (4) reported that PD patients exhibit problems in balance skills when their executive function is more compromised. Dahdal et al. (5) reported that, in PD patients, precision finger movements are more impaired when mild cognitive impairment is also present. In stroke patients, pyramidal weakness and cognitive disturbances often coexist. Thus, tasks that require both cognitive and motor skills have been used in some studies for the purpose of rehabilitation and prediction of functional prognosis after stroke (6, 7). Because motor and non-motor symptoms also coexist in PD patients, such integrative and comprehensive approach is important to the understanding of the real-life conditions of PD patients.

In daily life, cognitive and motor functions rarely operate in isolation. Even very simple actions may be preceded by decision processes to select an action from alternative options and by preparatory processes to generate purposeful movement. We are often required to execute optimal decisions and actions in a timely manner to adapt to a dynamically changing environment. For instance, when driving a car, one must perform a visual analysis of the information from traffic signs, other vehicles, and pedestrians to make adaptive decisions such as whether to apply pressure on the brake or accelerator pedals, or to turn the steering wheel. The basal ganglia and the dopamine system are known to play important roles in integrating cognitive and motor processing in the brain (8). Thus, it is plausible that various daily-life problems in PD patients are caused by disrupted interaction of cognitive and motor skills due to dopamine depletion and disintegrated cortico-basal ganglia circuits.

**Figure 1:**
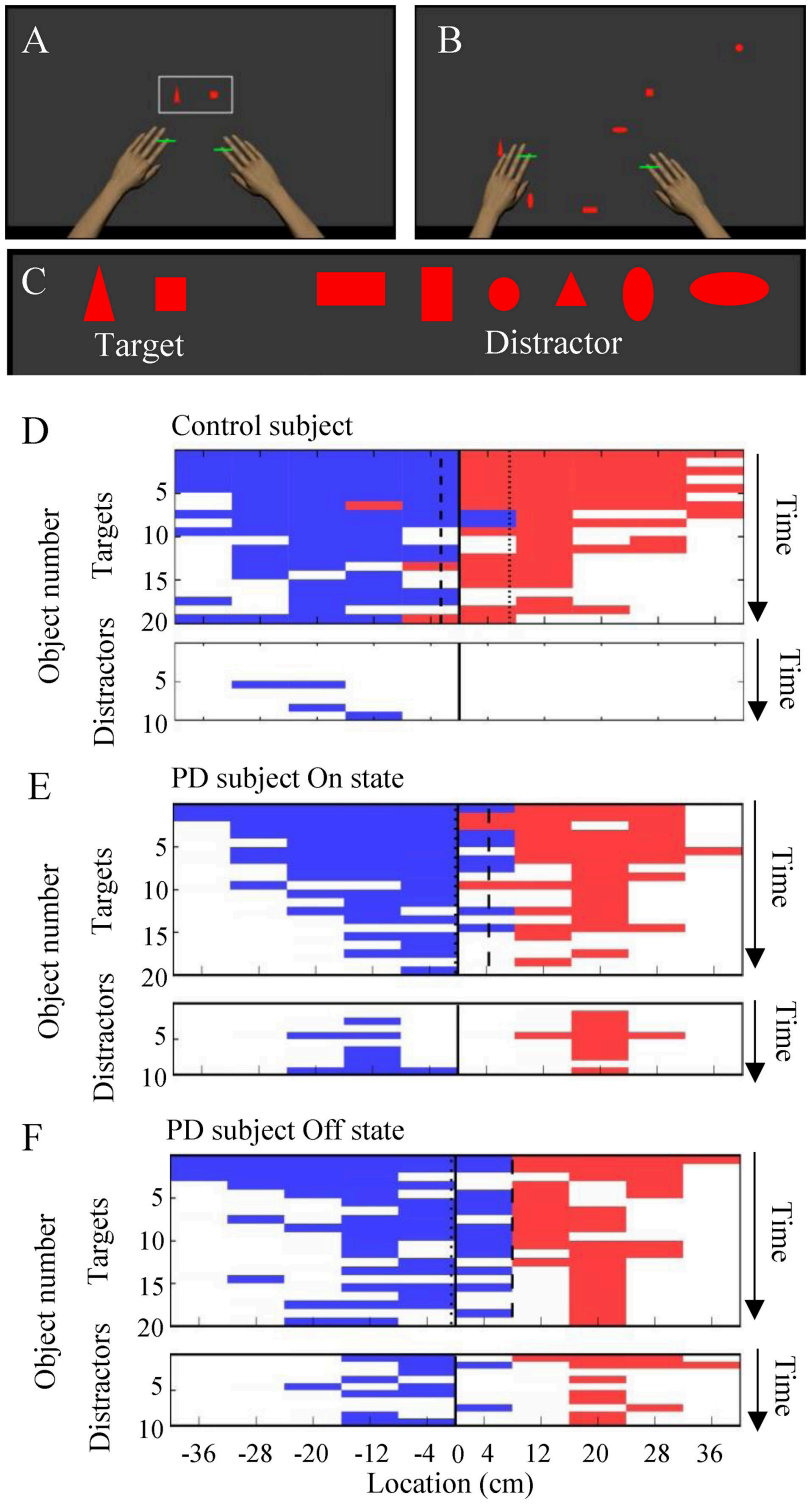
The object hit and avoid task and the results from exemplar subjects. **(A–C)** Virtual reality set-up. Two green cursors indicate the position of the middle fingers, which moved contingently with hand movement. The shapes of hit targets were displayed at the beginning of the task **(A)**. Targets and distractors fell down from the top of the screen **(B)**. Objects included two target shapes and six distractor shapes **(C)**. **(D–F)** Task performance results from exemplar subjects (**D**, control; **E**, PD in On state; **F**, PD in Off state; **E**,**F** are from the same subjects). The objects hit by the left and right hands are plotted in blue and red, respectively, at specific time (vertical axis) and at the specific spatial bin (horizontal axis). The target and distractor objects are plotted in the upper and lower panels, respectively. White areas indicate objects that were not hit.

The robotic manipulandum device allows for flexible visual presentation on an LCD screen and multi-joint free arm movement in two-dimensional space (Figure 1A). The device provides an excellent environment in which to study the interactions between voluntary motor control and various cognitive factors including visuospatial attention, working memory, and executive function. Previously, the robotic manipulandum device has been used to assess several clinical conditions including stroke and traumatic brain injuries (9–11), but it has not been used to assess PD patients.

The object hit and avoid task allows for testing of visual discrimination, rapid motor decisions, and precision arm movement. The robotic manipulandum device provides quantitative measures of arm kinematics as well as cognitive performance simultaneously.

In the present study, we aimed to use a robotic manipulandum device to examine the relationship between conventional clinical evaluation scores and performance in a virtual reality complex task in PD patients. We hypothesized that impairments of motor and cognitive skills would be detected with high sensitivity using a realistic task under high cognitive and kinematic loads. We compared the results of PD patients and age-matched healthy subjects and examined the acute effect of levodopa in PD patients.

## Methods

### Participants

We recruited PD patients and age-matched control subjects to participate in the present study. PD patients were enrolled from the outpatient clinic as well as inpatient ward of Fukushima Medical University Hospital, from 2014 to 2018. The inclusion criteria for PD patients were (1) to meet the UK Parkinson's Disease Brain Bank criteria, (2) with the Hoehn-Yahr grade ≤ IV, and (3) without severe dyskinesias. Exclusion criteria were patients with (1) a Mini Mental State Examination (MMSE) score of <23, (2) other psychiatric or additional neurologic condition, and (3) orthopedics, osteoarthritis, or any other conditions that might influence upper extremities sensation and motor movement. The control subjects were healthy volunteers without a history of neuropsychiatric disorders. We applied the same exclusion criteria as those of PD patients to the control subjects.

During the study period, the PD subjects were instructed to take their PD medications regularly. Each PD subject underwent the following cognitive and motor assessments during the On state (1–2.5 h after having taken their medication), and motor assessments during the Off state (>5 h after having taken their medication). Off state in this study was defined practically with limited drug withdrawal time, because complete drug withdrawal would have caused too severe akinesia to perform the robotic task.

### Clinical Assessment

Each subject underwent neuropsychological assessment consisting of the MMSE (12), Frontal Assessment Battery (FAB) (13), Raven's Colored Progressive Matrices (RCPM), Rey-Osterrieth Complex Figure Test (ROCF), and Overlapping Figure Test (OFT) (14, 15). The PD patients also underwent a standard odor test (odor stick identification test for Japanese, OSIT-J). Each PD subject underwent the cognitive batteries in On state (1–2.5 h after having taken their medication). We evaluated the motor symptoms of PD in the On and Off states using the Movement Disorder Society–Unified Parkinson's Disease Rating Scale (MDS-UPDRS) part III (16).

### Experimental Setup

We used a bimanual KINARM exoskeleton robot (BKIN Technologies Ltd, Kingston, ON, Canada) to evaluate upper limb motion during an object hit and avoid task. Details of the robotic set-up have been reported previously (17, 18). Briefly, the participants sat in a modified wheelchair base, and their arms were fitted in supports that permitted movement in the horizontal plane only. The arm supports were adjusted such that the robot's linkages aligned with the subject's elbows and shoulders. The subjects received visual feedback from a virtual reality system that displayed the fingertip position and virtual objects in the same plane as the arms.

### Behavioral Task

Each PD patient performed an object hit and avoid task twice during the On and Off states on separate days. The control subjects were tested once without medication. The details of the object hit and avoid task have been described previously (11) (Figures 1A–C). Briefly, at the beginning of the task, subjects were presented two shapes on the screen. The subjects were instructed to hit these two shapes (“targets”) away from them and avoid all other shapes (“distractors”). The subjects could use both hands, which were represented by horizontal paddles. The assignment for target/distractor shapes was randomized across sessions. Both targets and distractors fell from one of 10 locations that were placed 8 cm apart along the top of the screen (virtual bins). A total of 30 objects (20 targets and 10 distractors) were released from each bin (200 targets and 100 distractors in total). Objects were released from all 10 bins before a bin was reused. The objects dropped at an increasing rate, thus it moved at ~10 cm/s at the beginning of the task and increased to ~50 cm/s by the end of the task. The position of the objects and the hand position were recorded at 200 Hz. The task took a little over 2 min to complete. Targets hit by a paddle were knocked away and haptic feedback of the contact was provided by the robot, whereas distractors simply passed through the paddle to provide immediate feedback that it was a distractor. Every effort was made to ensure that the subjects understood the task instructions. The operators obtained verbal confirmation that the subjects understood which target to hit when presenting the target objects before starting the task.

### Data Analysis

The data from the bimanual KINARM exoskeleton robot was primarily analyzed by Dexterit-E Explorer version 1.4.0 (BKIN Technologies Ltd, Kingston, ON, Canada). Hand speeds were filtered using a sixth-order double-pass Butterworth filter with cut-off frequency of 10 Hz. We used four measurements to quantify the performance of the object hit and avoid task that were used in previous studies (9, 11).

1. Hand speed (cm/s). The robot measured the hand speed in every 5 ms for each hand. The average hand speed over the course of the task was calculated for each hand separately. We also calculated the grand average of hand speed for both hands.
2. Movement area (cm^2^). Movement area was the areas of space used by each hand during the task, which was computed as the area of the convex hull, i.e., a complex polygon that captures the boundaries of the movement trajectories of each hand (9). The robot measured the movement area for each hand separately. We also obtained the sum of the movement areas of both hands.
3. Targets hit score (%). The robot counted the number of target objects hit by the paddle over the course of the task. The proportion of the successful hits of 200 target objects was calculated and reported as the target hit score.
4. Distractor proportion (%). The robot counted the number of distractor objects that were hit by the paddle over the course of the task. The proportion of the erroneous distractor hits of the total number of objects hit (targets + distractors) was calculated and reported as distractor proportion.

### Statistical Analysis

We compared the four measurements (hand speed, movement area, targets hit score, and distractor proportion) between groups (PD On vs. Off using a paired *t*-test, and PD On vs. control and PD Off vs. control) using a *t*-test with Bonferroni correction. We then performed univariate linear regression of the target hit score and distractor proportion in the On and Off states. These dependent variables were regressed by the following variables: MDS-UPDRS part III score, hand speed, and movement area. The β values were tested against a null hypothesis (β = 0) by an *F* test.

To evaluate the influence of the cognitive factor on the robotic game performance, we conducted a median split of the PD cohort based on the MMSE score. The target hit score and distractor proportion were compared across three groups (PD with higher MMSE, PD with lower MMSE, and controls) by using a one-way analysis of variance (ANOVA). We also conducted Tukey HSD for *post-hoc* tests.

The data were analyzed using SPSS for Windows, version 23.0 (SPSS Inc., Illinois, United States). In all tests, a value of *p* < 0.05 was considered to be statistically significant.

## Results

We recruited 26 PD patients and 14 age-matched control subjects in the present study. We found no significant differences in age, education, or the scores of all the cognitive tests between the two groups (*p* > 0.05, *t*-test; Table 1). The MDS-UPDRS part III score was significantly different between On and Off states, as expected (*p* < 0.001). The OSIT-J score was significantly lower in PD patients than in the control group (*p* = 0.002, one-tailed *t*-test).

Table 1Demographics, clinical features, and data of robotic object hitting game.

**Table:** 

	**PD (*n* = 26)**	**Control (*n* = 13)**	***p*-value (PD vs. Control)**
Age (years)	63.8 ± 11.1	67.4 ± 9.1	0.297
Education (years)	13.3 ± 2.8	12.9 ± 2.0	0.830
Disease duration (years)	6.3 ± 4.2	—	—
LEDD (mg/day)	651.5 ± 423.1	—	—
H & Y stage	2.61 ± 0.57	—	—
MMSE	28.3 ± 2.3	28.4 ± 1.7	0.944
FAB	15.3 ± 3.0	16.8 ± 1.3	0.095
RCPM	28.6 ± 5.4	29.4 ± 4.3	0.615
ROCF	21.1 ± 9.1	23.9 ± 8.2	0.352
OFT	32.8 ± 5.7	33.6 ± 8.7	0.721
OSIT-J	4.85 ± 3.0	8.4 ± 2.3	<0.001

**Table:** 

	**PD**		***p*****-value (ES/power)**	**Control**	***p*****-value (ES/power)**	
	**Off**	**On**	**On vs. Off**		**PD**_****Off****_ **vs. Control**	**PD**_****On****_ **vs. Control**
MDS-UPDRS part III	35.0 ± 19.7	22.7 ± 18.4	<0.001^**^ (1.16/0.99)	—	—	—
Hand speed (cm/s)	10.9 ± 4.2	12.6 ± 4.8	0.002^**^ (0.67/0.79)	19.0 ± 5.3	<0.001^**^ (1.72/0.99)	0.001^**^ (1.25/0.89)
Movement area (cm^2^)	1134.9 ± 457.7	1288.9 ± 503.1	0.031 (0.45/0.41)	2002.3 ± 662.0	<0.001^**^ (1.63/0.99)	<0.001^**^ (1.27/0.91)
Target hit score (%)	51.6 ± 13.7	54.5 ± 11.4	0.061 (0.38/0.3)	63.3 ± 8.8	0.002^**^ (1.13/0.68)	0.012^*^ (0.87/0.56)
Distractor proportion (%)	21.3 ± 11.4	19.7 ± 10.0	0.362 (0.18/0.07)	13.2 ± 6.2	0.007^**^ (0.95/0.48)	0.035 (0.72/0.39)

Values are presented as a mean ± standard deviation. The demographics and the scores of cognitive batteries were compared between the Parkinson's disease (PD) and control groups. The Movement Disorder Society—Unified Parkinson's Disease Rating Scale (MDS-UPDRS) part III score and the four kinematic measures of the robotic object hitting game were compared between the Off and On levodopa conditions in the PD patients by paired t-test, between PD_Off_ and controls, and between PD_On_ and controls, separately. ^**^ and ^*^ represent statistical significance at p < 0.01 and p < 0.05, respectively (multiple comparisons corrected by Bonferroni method). Values in the brackets indicate Cohen's d effect size and power. LEDD, Levodopa Equivalent Daily Dose; H & Y, Hoehn-Yahr scale; MMSE, Mini Mental State Examination; FAB, Frontal Assessment Battery; RCPM, Raven's Colored Progressive Matrices; ROCF, Rey-Osterrieth Complex Figure; OFT, Overlapping Figure Test; OSIT-J, Odor Stick Identification Test for the Japanese; ES, Cohen's d effect size.

**Figure 2:**
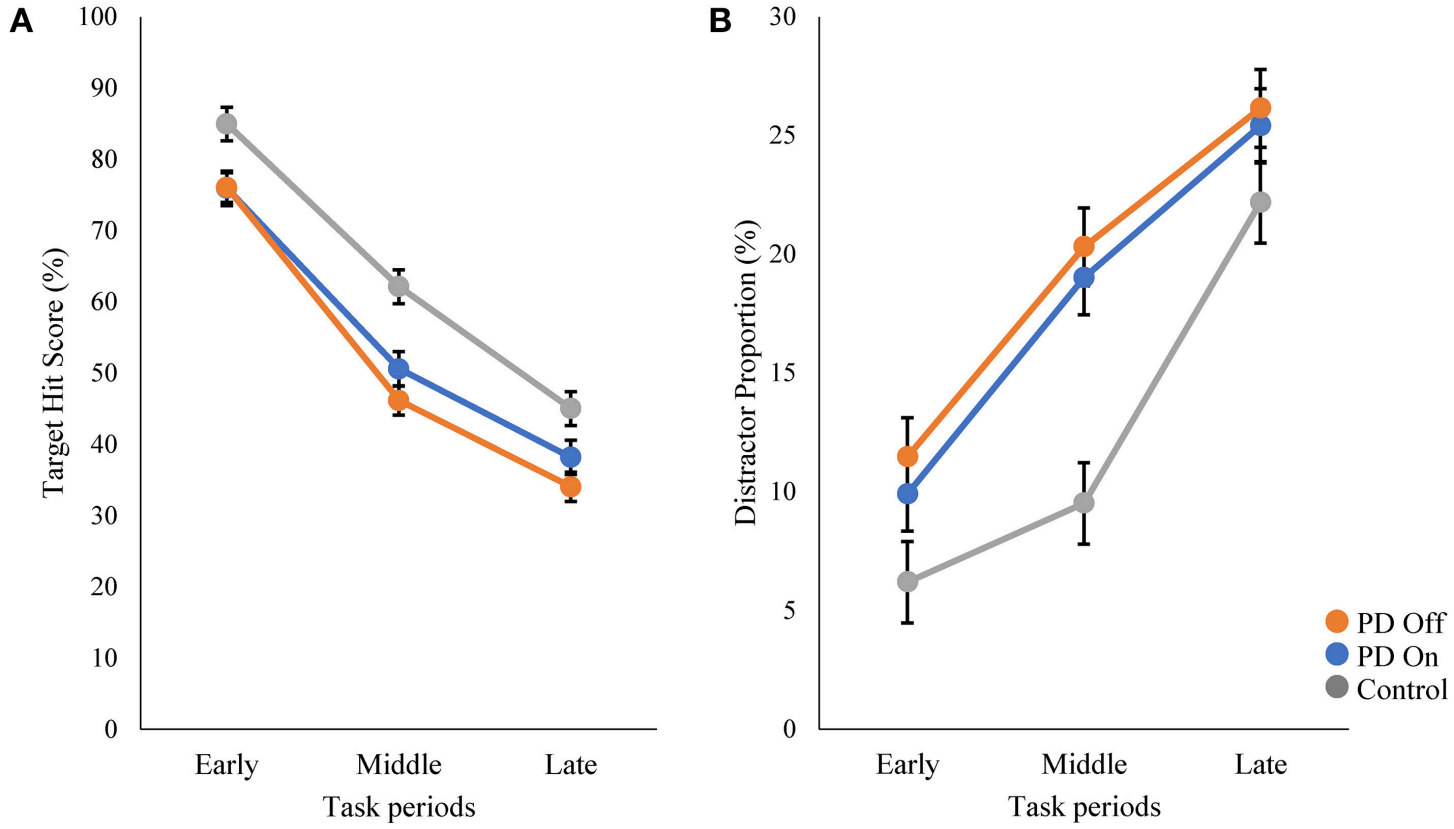
Comparisons of target hit score and distractor proportion in three task periods. The whole task session with 300 falling objects was divided into three periods (early, middle, and late; 100 objects in each period). The speed of target and distractor progressively increased during these three periods. The scores of target hit **(A)** and distractor proportion **(B)** are plotted for each period.

We compared the performance on the robotic object hit and avoid task between the PD and control groups (Figures 1D–F and Supplementary Video). The control subjects hit significantly more targets than did the PD patients (*p* < 0.001, one-tailed *t*-test; Table 1). In contrast, the control subjects hit significantly fewer distractors than did the PD patients (*p* < 0.005, *t*-test). Between the On and Off states of PD patients, neither the target hit score nor distractor proportion changed significantly (*p* > 0.016, paired *t*-test). Because the pace of object presentation increased with time, the task increased in difficulty toward the end (Figure 2). The two-way ANOVA (time and group) revealed a significant main effect of time for both target hit score [*F*_(2, 126)_ = 323.4, *p* < 0.001] and distractor proportion [*F*_(2, 126)_ = 50.83, *p* < 0.001]. The group main effect was also significant for target hit score [*F*_(2, 63)_ = 4.624, *p* = 0.013] and the distractor proportion [*F*_(2, 63)_ = 3.703, *p* = 0.03]. Tukey HSD tests revealed that the control group hit more targets and less distractors than PD in Off states (*p* < 0.05). There was no significant interaction effect between time and group (*p* > 0.05). These results indicate that the performance of the PD patients on the hit and avoid task was poorer than that of the control subjects, as they hit fewer targets and more distractors.

We examined the effect of levodopa on kinematic measures and found that the average hand speed increased significantly with the use of levodopa in PD patients (*p* < 0.01, paired *t*-test, Table 1). Because hand motor symptoms are often asymmetric, we conducted two-way ANOVA (Affected side × On-Off state). The analysis revealed significant main effects of both factors (*p* < 0.01), but their interaction was insignificant (*p* = 0.1; Supplementary Table 1). Hand kinematics also differed significantly between the PD and control groups; the PD patients were slower and moved their hands within a smaller area than the controls did (*p* < 0.001, *t*-test with Bonferroni correction; Table 1).

**Figure 3:**
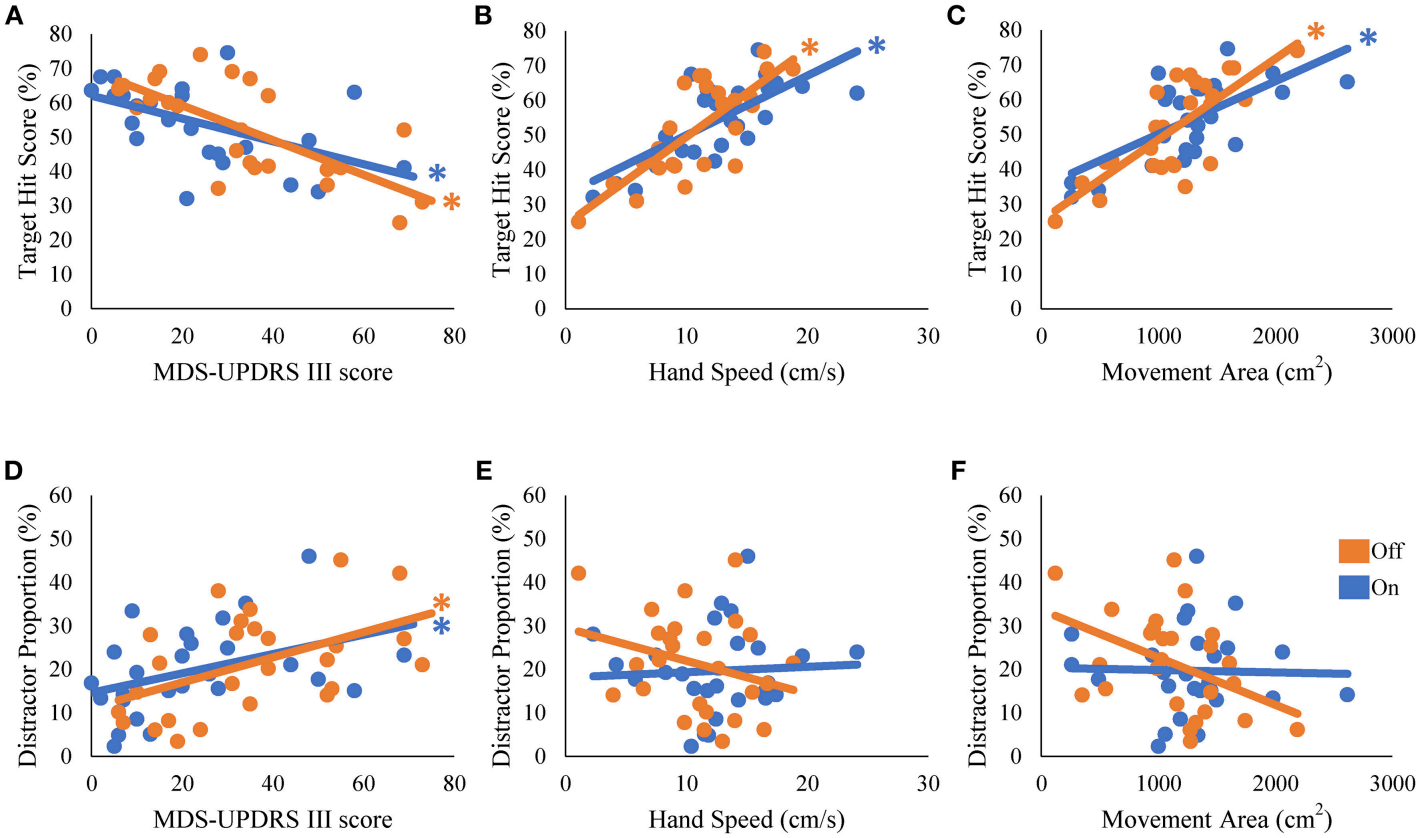
Univariate regression of the performance scores of the robotic game. The scores of target hit **(A–C)** and the distractor proportion **(D–F)** were regressed with disease severity (MDS-UPDRS part III) **(A,D)**, hand speed **(B,E)**, and movement area **(C,F)**. * represents statistical significance of the β at *p* < 0.05.

We then examined how well the task performance could be predicted by disease severity and hand kinematics in the PD group. The target hit score correlated negatively with the MDS-UPDRS part III score, and positively with hand speed and movement area; patients with more severe symptoms (i.e., a higher MDS-UPDRS part III score) hit fewer targets in the On [β = −0.54, *t*_(24)_ = −3.11, *R*^2^ = 0.29, *p* = 0.005] and Off states [β = −0.73, *t*_(24)_ = −5.21, *R*^2^ = 0.29, *p* < 0.001; Figure 3A]. Patients with more rapid hand speed and larger movement area hit more targets in the On [β = 0.71, *t*_(24)_ = 4.99, *R*^2^ = 0.51, *p* < 0.001 and β = 0.67, *t*_(24)_ = 4.39, *R*^2^ = 0.45, *p* < 0.001, respectively; Figures 3B,C] and Off states [β = 0.78, *t*_(24)_ = 6.06, *R*^2^ = 0.61, *p* < 0.001 and β = 0.77, *t*_(24)_ = 5.98, *R*^2^ = 0.60, *p* < 0.001, respectively; Figures 3B,C]. The number of erroneous distractor hits exhibited a significantly positive correlation with MDS-UPDRS part III score [On state, β = 0.41, *t*_(24)_ = 2.22, *R*^2^ = 0.17, *p* = 0.036; Off state, β = 0.50, *t*_(24)_ = 2.81, *R*^2^ = 0.25, *p* = 0.01; Figure 3D]. However, distractor proportion did not correlate significantly with hand speed or movement area (*p* > 0.05; Figures 3E,F). Over all, target hit score was regressed well with kinematic parameters, but fitting of the distractor proportion was generally poor. These results suggest that the kinematic capacity of PD patients is an important predictor for the performance of target hit, but not of distractor avoidance.

**Figure 4:**
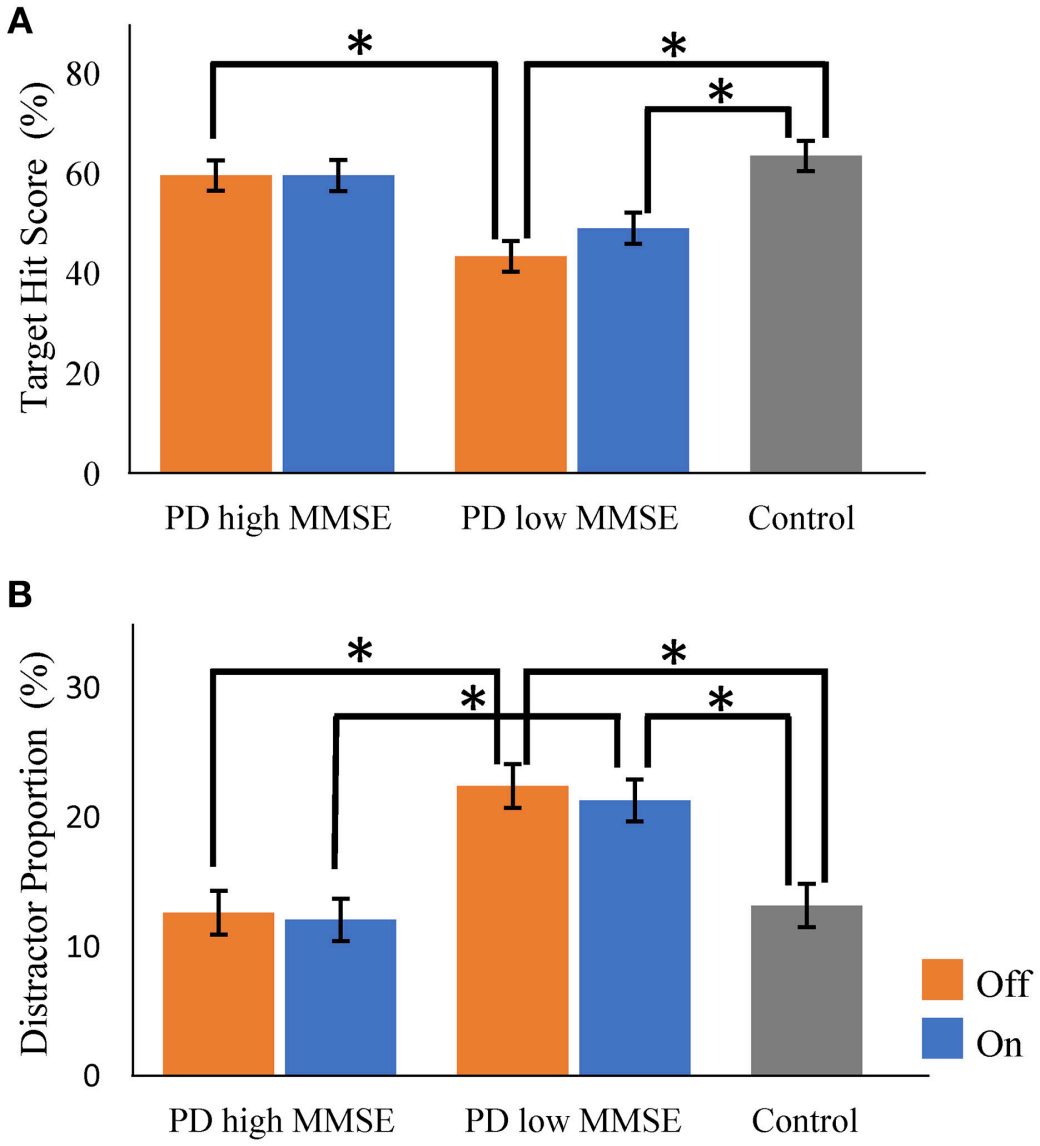
Comparisons of target hit score and distractor proportion in Parkinson's disease patients with higher and lower Mini Mental State Examination (MMSE) score (median split). There were significant group differences for both the target hit score **(A)** and the distractor proportion **(B)** (*p* < 0.001, one-way ANOVA). Parkinson's disease (PD) with higher MMSE scores and control subjects hit more targets than did PD with lower MMSE scores **(A)**. PD with lower MMSE scores hit more distractors than the PD with higher MMSE scores and control subjects did **(B)**. Orange bars, PD in Off state; Blue bars, PD in On state. Gray bars, control. Error bars, standard error of mean. ^*^ Represents significance at *p* < 0.05 by Tukey HSD tests.

To examine the influence of global mental state on the performance of hit and avoid task, we conducted a median split analysis by dividing PD patients into two subgroups based on MMSE score (Figure 4). A one-way ANOVA of the target hit score for group with five levels (High MMSE On, High MMSE Off, Low MMSE On, Low MMSE Off, and Control) revealed a significant main effect for Group [*F*_(4, 61)_ = 8.876, *p* < 0.001; Figure 4A]. PD patients with higher MMSE scores hit more targets than PD patients with lower MMSE scores did in Off state (*p* < 0.05, Tukey HSD). In the On state, PD patients with higher MMSE scores showed tendency to hit more targets than PD patients with lower MMSE scores did (*p* = 0.08, Tukey HSD). In addition, the control subjects hit more targets than the PD with lower MMSE scores did in both On and Off states (*p* < 0.05, Tukey HSD). A one-way ANOVA for the distractor proportion also revealed a significant main effect of group [*F*_(4, 61)_ = 8.562, *p* < 0.001; Figure 4B]. *Post-hoc* Tukey HSD tests revealed that PD patients with higher MMSE scores hit fewer distractors than did PD with lower MMSE scores in both the On and Off states. Additionally, the control group hit fewer distractors than the PD with lower MMSE scores did in both the On and Off states (*p* < 0.05). Among the five cognitive batteries, the results of the MMSE, FAB, and RCPM exhibited strong correlations each other (ρ ≈ 0.4–0.8, *p* < 0.05; Table 2). By using median split based on the FAB and RCPM scores, the above results based on the MMSE score (Figure 4) were generally reproduced (Supplementary Figure 1). In separate correlation analysis, the MMSE, FAB, RCPM scores exhibited a strong correlation with the distractor proportion (Table 3). In contrast, the hand kinematic parameters (speed and movement area) exhibited a strong correlation with the target hit score, but not with the distractor proportion. Taken together, the results suggest that global cognitive capacity has significant influences on the ability to hit targets and avoid distractors.

**Table 2:** Correlation matrix of the scores of cognitive batteries.

**Cognitive test batteries**	**MMSE**	**FAB**	**RCPM**	**Rey**	**OFT**	**OSIT-J**
MMSE	1.000					
FAB	0.647^**^	1.000				
RCPM	0.769^**^	0.427^*^	1.000			
ROCF	0.496^*^	0.163	0.386	1.000		
OFT	0.481^*^	0.293	0.447^**^	0.335	1.000	
OSIT-J	0.284	0.186	0.279	0.253	0.209	1.000

Correlation coefficients are Spearman's ρ. ^**^ and ^*^ represent statistical significance at p = 0.01 and 0.05, respectively (2-tailed). MMSE, Mini Mental State Examination; FAB, Frontal Assessment Battery; RCPM, Raven's Colored Progressive Matrices; ROCF, Rey-Osterrieth Complex Figure Test; OFT, Overlapping Figure Test; OSIT-J, Odor Stick Identification Test for the Japanese. Correlation between variables using Spearman's ρ. ^**^ and ^*^ represent significance level at p = 0.01 and 0.05, respectively (2-tailed).

**Table 3:** Correlation between task performance (target hit score and distractor proportion) and other variables.

	**Target hit score**				**Distractor proportion**			
	**OFF**		**ON**		**OFF**		**ON**	
	**ρ**	***p***	**ρ**	***p***	**ρ**	***p***	**ρ**	***p***
MDS-UPDRS III	−0.729	<0.001^**^	−0.536	0.05^*^	0.497	0.01^*^	0.412	0.036^*^
Hand Speed	0.778	<0.001^**^	0.714	<0.001^**^	−0.277	0.17	0.059	0.776
Movement area	0.774	<0.001^**^	0.667	<0.001^**^	−0.434	0.027^*^	−0.029	0.890
MMSE	0.533	0.005^**^	0.472	0.015^*^	−0.616	0.001^**^	−0.632	0.001^**^
FAB	0.379	0.056	0.257	0.206	−0.581	0.002^**^	−0.424	0.031^*^
RCPM	0.443	0.024^*^	0.270	0.182	−0.512	0.007^**^	−0.550	0.004^**^
Rey	0.323	0.108	0.411	0.037^*^	−0.254	0.211	−0.421	0.032^*^
OFT	0.444	0.023^*^	0.537	0.005^**^	−0.411	0.037^*^	−0.371	0.062
OSIT-J	0.389	0.05^*^	0.363	0.069	−0.114	0.579	−0.205	0.315

ρ is Pearson's correlation coefficient for the Movement Disorder Society—Unified Parkinson's Disease Rating Scale (MDS-UPDRS III), hand speed, and movement area, and it is Spearman's correlation coefficient for Mini Mental State Examination (MMSE), Frontal Assessment Battery (FAB), Raven's Colored Progressive Matrices (RCPM), Rey-Osterrieth Complex Figure Test (ROFT), Overlapping Figure Test (OFT), and Odor Stick Identification Test for the Japanese (OSIT-J). ^**^ and ^*^ represent statistical significance at p = 0.01 and p = 0.05, respectively (2-tailed).

## Discussion

We examined 26 PD patients and 14 control subjects by using a robotic object hitting game that required visual discrimination of the objects and accurate motor control. The performance of the PD patients was generally poorer than that of controls. The clinical score of motor symptoms (i.e., MDS-UPDRS part III) of PD patients predicted the number of targets hit and distractors hit (Table 2). The numbers of hitting targets and distractors also depended on the individual MMSE score; PD patients with low MMSE scores hit fewer targets and more distracters than did PD patients with high MMSE scores and controls (Figure 4). These results suggest functional interactions between the cognitive system that discriminates stimuli and makes decisions, and the motor system that controls rapid and accurate sequential hand responses.

One of the advantages of using the robotic device is its precise kinematic measurements. Accurately measured hand speed and movement area predicted the total number of targets hit. The robotic device also detected the asymmetry of the motor symptoms; the more affected hand was slower and hit less number of targets than the less affected hand did (Supplementary Table 1). The effect of levodopa on the kinematic measurements was also evident. Motor improvement in response to levodopa is a hallmark of PD. With the advances of sensor technologies, the increasing number of studies report quantitative evaluation of drug effects on kinematics of movement disorders (19–24). Movement disorders of basal ganglia and cerebellar origins are characterized by disturbances in accurate and smooth execution of voluntary movement. The present study demonstrates the robotic manipulandum device is useful in the evaluation of movement disorders with its precise measurements of the upper extremities during free and natural movements.

In addition to motor control, the present task required cognitive resources, including visual discrimination, general attention, and inhibitory control (11). Indeed, we found that the MMSE and FAB scores were significant predictors of target hit score and distractor proportion (Figure 4 and Supplementary Figure 1). The scores of MMSE, FAB and RCPM correlated with each other (Table 2), suggesting an underlying general factor of intelligence (25). These results indicate that global cognitive capacity is a factor that explains the between-subject variability in game performance in PD patients.

Olfactory dysfunction as measured by OSIT-J showed insignificant correlation with the cognitive screening tests (Table 2). OSIT-J showed weak but significant correlation with the number of the target hit in the OFF state (Table 3). Because hyposmia is known to predict cognitive decline in PD in a few years of time (26), OSIT-J score may be predicting future performance of the hit and avoid task.

Single-dose levodopa did not influence cognitive skills to the same extent as it did motor control. Clinical research on the dopaminergic influence on cognitive performance remains controversial (27, 28). In a physiological study, using a robotic manipulandum device in macaque monkeys, dopamine neurons were demonstrated to generate teaching signals that guide visually triggered reaching movement (29). Thus, the dopamine system appears to structure the temporal aspect of motor planning. Further study is needed to clarify the effects of dopaminergic treatment on cognitive function in PD patients.

Motor dysfunction in PD is generally attributed to dopamine deficiency in the basal ganglia. Cognitive impairments in PD are suggested to be related to the dysfunction of the fronto-basal ganglia circuit (30–34). There are some reports that suggest contribution of the cerebellum to cognition (35). However, PD patients manifest neither cerebellar symptoms nor cerebellar atrophy. Thus, we believe that the cerebellar contribution in both motor and cognitive domains of PD patients is minimal. In this respect, the patients with multiple system atrophy (MSA), who suffer from both basal ganglia and cerebellar degeneration, would be interesting. Robotic object hitting game with MSA patients would test for the cerebellar-basal ganglia interaction and their contribution to cognitive and motor skills.

The present study has several limitations. First, the number of subjects in the present study was relatively small and the results of this pilot cross-sectional study should be confirmed in future studies with a larger scale. Second, we used a within-subject design for levodopa for motor performance including the robotic object hitting game and the MDS-UPDRS, but the cognitive batteries were tested only in the On state. Assessment of the effects of levodopa on the scores of cognitive batteries is warranted for future testing using a within-subject design.

In summary, we demonstrated dopaminergic enhancement of kinematic features during a dynamic and multifactorial game setting. The global cognitive capacity predicted the ability to hit targets and avoid distractors. The current device-aided set-up that allows for objective and quantitative measurements of cognitive and motor skills would be useful in the assessment of therapeutic effects in clinical trials, estimating daily life activities, and predicting disease prognosis.

## Ethics Statement

This study was carried out in accordance with the Declaration of Helsinki. The protocol was approval by Ethics Committee of Fukushima Medical University. Informed Consent was obtained in a written document from all subjects.

## Author Contributions

SK and YU designed the study. WW, SK, KA, and F-YC conducted the experiments. WW and SK analyzed the data and drafted the manuscript.

### Conflict of Interest Statement

The authors declare that the research was conducted in the absence of any commercial or financial relationships that could be construed as a potential conflict of interest.

## Abbreviations

PD: Parkinson's disease
MMSE: Mini Mental State Examination
FAB: Frontal Assessment Battery
RCPM: Raven's Colored Progressive Matrices
ROCF: Rey-Osterrieth Complex Figure Test
OFT: Overlapping Figure Test
OSIT-J: Odor Stick Identification Test for the Japanese
MDS-UPDRS: Movement Disorder Society—Unified Parkinson's Disease Rating Scale.
