# Supplementary material for: Assessment of Cognitive and Motor Skills in Parkinson's Disease by a Robotic Object Hitting Game
Source: Front Neurol. 2019 Jan 28;10:19. doi: 10.3389/fneur.2019.00019 (PMC6360146; doi:10.3389/fneur.2019.00019)
Supplement: Supplementary file 2 [file Data_Sheet_1.docx]

Supplementary Material

Assessment of Cognitive and Motor Skills in Parkinson’s Disease by A Robotic Object Hitting Game

**Winnugroho Wiratman, Shunsuke Kobayashi*, Fang-Yu Chang, Kohei Asano, Yoshikazu Ugawa**

*** Correspondence:** Corresponding Author: [skoba-tky@umin.net](mailto:skoba-tky@umin.net)

1. **Methods**

We judged which hand was more affected by Parkinsonism based on Movement Disorder Society – Unified Parkinson’s Disease Rating Scale (MDS-UPDRS) part III motor scores; we calculated the subtotal scores of subsections (3.3, 3.4, 3.5, and 3.6) that measure rigidity and dysdiadochokinesis of the upper extremities, and compared between the left and right hands. The hand with the higher score was judged to be more affected. We evaluated the four measurements (hand speed, movement area, targets hit score, and distractor proportion) at each hand in On and Off states. For statistics, we conducted two-way analysis of variance (ANOVA) with factors of the affected side and On-Off states. We used Bonferroni corrected post-hoc paired *t*-test.

1. **Results**

We found asymmetric hand involvement in 21 patients (right, 13; left, 8). In five patients, both hands were equally affected, hence excluded from the analysis. The data from the remaining 21 patients were submitted to the two-way ANOVA, and the results are shown in supplementary Table 1. For both hand speed and movement area, the main effects of the affected side and On-Off state were significant. For target hit score, the main effect of the affected side was significant; i.e., the more severely affected hand hit less number of targets. There was no significant interaction effect between the two factors in any of the four measurements (hand speed, movement area, targets hit score, and distractor proportion).

**Supplementary Table 1.** Two-way analysis of variance (ANOVA, Affected side ×On-Off state) on the four behavioral measurements of the robotic game.

|  | Affected side main factor | | On-Off state main factor | | Affected side × On-Off state interaction | |
| --- | --- | --- | --- | --- | --- | --- |
|  | *F* (1,20) | *p* | *F* (1,20) | *p* | *F* (1,20) | *p* |
| Hand speed (cm/s) | 9.591 | 0.006** | 17.835 | <0.001** | 2.954 | 0.101 |
| Movement area (cm^2^) | 4.746 | 0.041* | 8.676 | 0.008** | 0.163 | 0.691 |
| Target hit score (%) | 4.883 | 0.039* | 2.944 | 0.102 |  |  |
| Distractor proportion (%) | 0.291 | 0.595 | 0.831 | 0.373 | 0.107 | 0.747 |

|  | On | | | Off | | |
| --- | --- | --- | --- | --- | --- | --- |
|  | More affected hand | Less affected hand | Post-hoc Paired *t*-test | More affected hand | Less affected hand | Post-hoc Paired *t*-test |
| Hand speed (cm/s) | 10.5 ± 4.2 | 13.6 ± 5.6 | 0.003** | 8.9 ± 3.6 | 10.9 ± 4.5 | 0.024* |
| Movement area (cm^2^) | 554.4 ± 278.9 | 666.5 ± 277.7 | 0.036 | 474.7 ± 205.6 | 572.8 ± 252.8 | 0.077 |
| Target hit score (%) | 50.5 ± 14.2 | 56.2 ± 12.6 | 0.044 | 47.6 ± 10.9 | 52.5 ± 17.6 | 0.55 |
| Distractor proportion (%) | 9.5 ± 4.7 | 9.0 ± 4.9 | 0.089 | 10.1 ± 5.1 | 9.9 ± 5.7 | 0.846 |

The four kinematic measures of the robotic game were tested by 2-way ANOVA (Affected side × On-Off state). Post-hoc pair-wise comparisons were done between more affected and less affected hands and between Off and On states. * and ** represent statistically significance at *p* < 0.05 and *p* < 0.01, respectively (Bonferroni correction). Values are presented as a mean ± standard deviation.


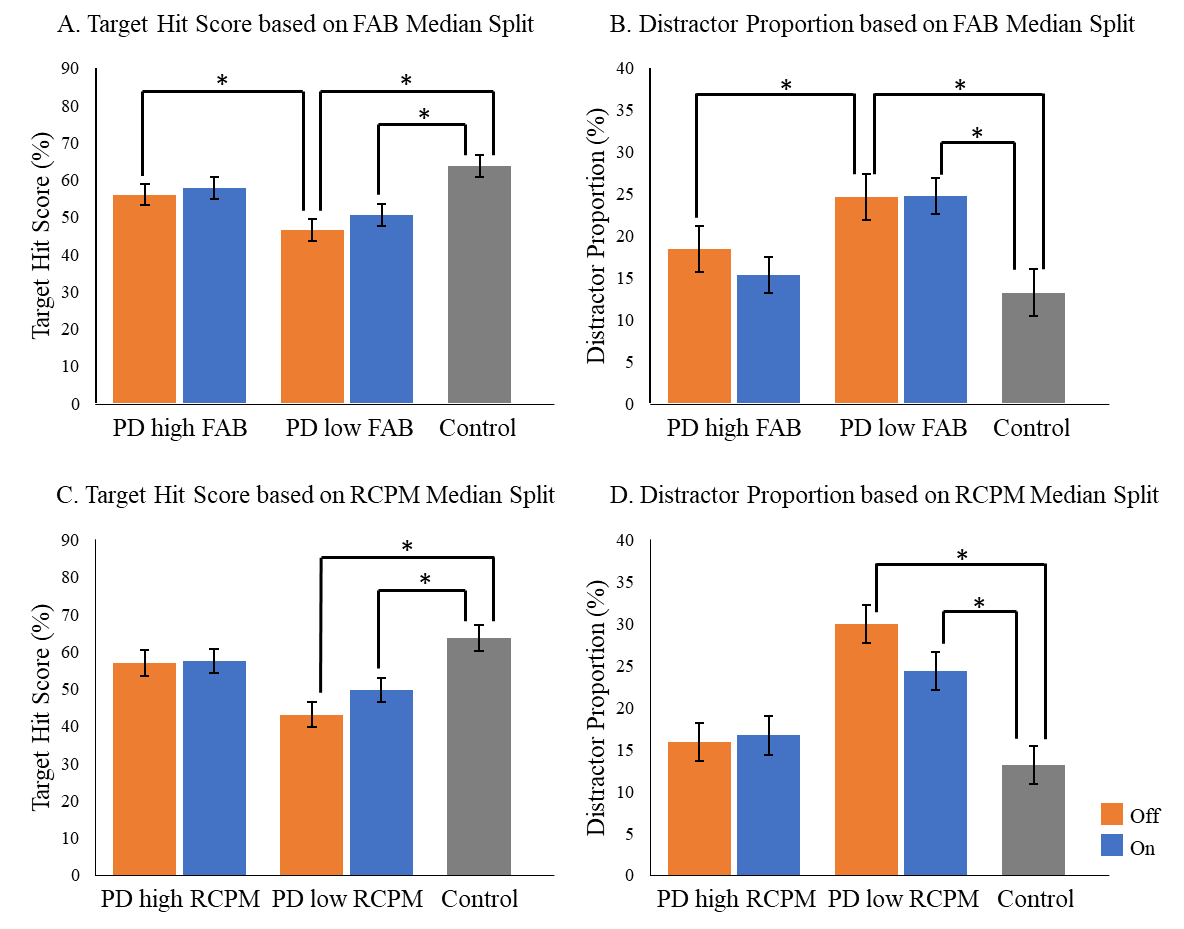


**Supplementary Figure 1.** Target hit score and distractor proportion in relation with the performance in FAB and RCPM. PD patients were divided into two groups by median split based on FAB (A, B) and RCPM (C, D). There were significant group main effect for both target hit score (A, C) and distractor proportion (B, D) (*p* < 0.001, one-way ANOVA). Orange bars, PD in Off state; Blue bars, PD in On state; Grey bars, control subjects. Error bars, standard error of the mean. * represent significance at *p* = 0.05 by Tukey’s post-hoc tests.
